# *PDCD1* (PD-1) is a direct target of *miR-15a-5p* and *miR-16-5p*

**DOI:** 10.1038/s41392-021-00832-9

**Published:** 2022-01-19

**Authors:** Alexey Palamarchuk, Liudmyla Tsyba, Luisa Tomasello, Yuri Pekarsky, Carlo M. Croce

**Affiliations:** Department of Cancer Biology and Genetics, Comprehensive Cancer Center, The Ohio State University, Columbus, OH 43210 USA

**Keywords:** Haematological cancer, Non-coding RNAs

**Dear Editor,**

Programmed cell death protein 1 (PD-1; encoded by the *PDCD1* gene), mostly expressed on activated T cells, is an important receptor in T-cell immunity.^1–3^ Together with programmed death-ligand 1 (PD-L1: encoded by the *CD274* gene) PD-1 acts as an inhibitor of T cell activity under normal conditions. In addition to T-cells PD-1 is abundantly expresses in some of B-cell malignancies, including Hodgkin’s Lymphoma and Follicular lymphoma.^1–3^ PD-1 expression was also detected in CLL.^1^ Notably, circulating T-cells in CLL patients express higher PD-1 levels than T-cells of healthy donors.^1–3^ Interestingly, various types of cancer express high levels of PD-L1 and are able to use PD-1/PD-L1 signaling to evade T cell immunity.^1–3^ Moreover, interruption of immune surveillance promotes cancer cell survival by exploiting PD-1/PD-L1 signaling.^1–3^ In recent years, many therapeutic antibodies against PD-1 and PD-L1 have been developed and have demonstrated promising results in clinical trials for various types of cancer.^1–3^ The highest response rate to PD-1 blockade was achieved in classical Hodgkin lymphoma.^1^ In recent years anti PD-1 antibodies (Opdivo and Keytruda), as well as anti PD-L1 antibodies (Tecentric and Imfinzi) disrupting PD-1–PD-L1 interaction, were FDA approved for treatment of a number of cancers. These include melanoma, kidney cancer, bladder cancer, lung cancer, Hodgkin’s lymphoma and others.^1–3^ *miR-15/16* is a key tumor suppressor microRNA cluster first identified as a target of 13q deletions in CLL.^4^ A recent report demonstrated that *miR-16* regulates PD-L1 expression in prostate and other cancers.^5^ *miR-15/16* are the first tumor suppressor microRNAs identified and alterations in *miR-15/16* provided the first evidence of the involvement of noncoding RNAs in cancer pathogenesis.^4^ Loss of *miR-15/16* is the most common genetic lesion in chronic lymphocytic leukemia, promoting overexpression of BCL2, resulting in leukemia.^4^ In addition to *BCL2*, *miR-15/16* cluster targets multiple oncogenes, including *ROR1*, *Cyclin D1*, and others. Thus, *miR-15/16* play an important role in many blood malignancies and solid cancers.^4^ For example, *miR-15/16* inhibit tumor progression by directly targeting MYCN in neuroblastoma; *miR-15/16* inhibit hepatocellular carcinoma progression by targeting FEAT through NF-κB signaling pathway.

Since *miR-15/16* target PD-L1 expression^5^ it is likely to regulate PD-1 – PD-L1 interaction. Thus, we thought that *PDCD1* might be also regulated by *miR-15/16*. To determine if this is the case, we analyzed 3′ UTR of *PDCD1* using TargetScan 7.2 software (http://www.targetscan.org). Remarkably we found that 3’ UTR of *PDCD1* contains a 28 bp DNA fragment containing eight overlapping *miR-15/16* target sites (exact match to positions 2–8 of the mature *miR-15a-5p* and *miR-16-5p*) (Fig. 1a). Thus, we proceeded to determine if *miR-15/16* target PDCD1 expression. To address this, we first checked the effect of overexpressed mimics for *miR-15a-5p* and *miR-16-5p* on PD-1 in HEK293 cells. Since HEK293 cells do not express any detectable endogenous *PDCD1*, we co-transfected HEK293 cells with pCMV-*PDCD1* (a mammalian expression vector containing full-length *PDCD1* cDNA including 3’ UTR, obtained from OriGene), and set of pre-miRNA mimics from ThermoFisher (pre-miR negative control, *pre-miR-15a-5p*, *pre-miR-16-5p* and *pre-miR-148a-3p*). These results revealed that co-expression of *PDCD1* and *miR-15a-5p* and *miR*-16-5p, but not *miR-148a-3p* (as a negative control) dramatically decreased PD-1 expression (Fig. 1b). In these experiments we routinely achieved ~70% transfection efficiency (Fig. 1c). To determine if these effects occur on translational level, we also measured the RNA expression of *PDCD1*, *miR-15a*, and *miR-16* (Fig. 1d, e). We found that *miR-15a* and *miR-16* expression decreased *PDCD1* mRNA expression by 33 and 22%, respectively (Fig. 1d), while *PDCD1* protein expression was decreased by 100 and 92% respectively (Fig. 1b). We concluded that *miR-15a-5p* and *miR-16-5p* target *PDCD1* expression mostly at protein level. To determine whether *PDCD1* is a direct target of *miR-15a-5p* and *miR-16-5p*, we performed luciferase reporter assays. HEK293 cells were co-transfected with constructs containing luciferase gene alone (psiCHECK2-empty) or fused with a 3’-UTR of *PDCD1* containing a 28 bp DNA fragment including eight overlapping *miR-15/16* target sites (psiCHECK2-*PDCD1* WT) and miR negative control (N.C.1), *miR-15a-5p* or *miR-16-5p* mimics. In addition, we used psiCHECK2-*PDCD1* MUT construct (psiCHECK2-*PDCD1* WT, lacking 28 nt-long region that contains 8 overlapping possible binding sites for *miR-15a-5p* and *miR-16-5p*). Fig. 1f (left) shows that *miR-15a-5p* and *miR-16-5p* expression did not affect the luciferase activity of psiCHECK2 empty vector. On the other hand, *miR-15a-5p* and *miR-16-5p* expression significantly decreased the luciferase activity of the psiCHECK2-*PDCD1* WT (Fig. 1f, middle). Co-transfecting *miR-15a-5p* and *miR-16-5p* and construct containing mutated form of *PDCD1* 3′-UTR (psiCHECK2-*PDCD1* MUT) completely negated this effect (Fig. 1f, right). These results confirmed that *miR-15a-5p* and *miR-16-5p* bound directly and specifically to its target sites within the 3′-UTR of *PDCD1*.

**Fig. 1:**
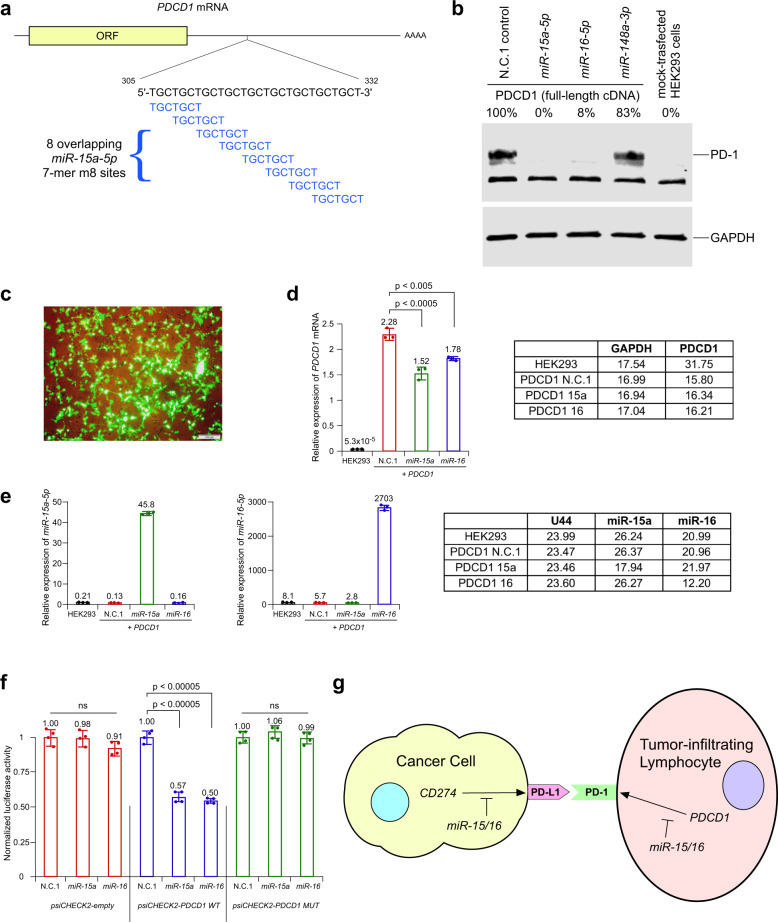
*miR-15/16* target *PDCD1* (PD-1) expression. **a** A 28 bp DNA fragment containing eight overlapping *miR-15/16* target sites in the 3′ UTR of *PDCD1*. **b** *miR-15/16* inhibit PD-1 protein expression. *miR-15a-5p* and *miR-16-5p* expression suppress the PDCD1 expression by targeting its 3’-UTR. Western blot data showed the protein level of overexpressed PDCD1 in HEK293 cells co-transfected with scrambled pre-miR negative control, *pre-mir-15a-5p*, *pre-miR-16-5p* and *pre-miR-148a-3p* mimics. GAPDH served as a loading control. **c** An example of transformation efficiency in experiments in HEK293 cells for western blot and Luciferase assays (as shown in **b**, **f**). **d**, **e** Results of real-time PCR experiments on RNAs isolated from HEK293 cells co-transfected with *PDCD1* construct and miR mimics (same as in **b**). **d** Results of real-time PCR using TaqMan probe for human *PDCD1* transcript. Average CT numbers are shown (right). **e** Results of real-time PCR using TaqMan probes for human *miR-15a-5p* (left) and *miR-16-5p* (middle). Average CT numbers are shown (right). **f** *PDCD1* is a direct target of *miR-15/16*. Renilla luciferase reporter assay showing the reporter expression in HEK293 cells co-transfected with wild-type 3′-UTR of *PDCD1* and mutant 3′-UTR of *PDCD1* along with scrambled negative control 1, *miR-15a* and *miR-16* mimics. *Renilla* luciferase activity was normalized to firefly luciferase activity. The normalized luciferase activities in HEK293 cells transfected with different psiCHECK2 constructs and scrambled negative control 1 (pre-miR-N.C.1) were set at 1 and relative luciferase activities of HEK293 cells co-transfected with each psiCHECK2 construct and *miR-15a* or *miR-16* mimics are shown. Two independent experiments were carried out in duplicates and data were presented as mean ± SD. **g** *miR-15/16* are key regulators of PD-1–PD-L1 interaction

PD-1, together with its ligand PD-L1 functions as a negative regulator of T-cell response in immune system. PD-1–PD-L1 interaction is a critical mechanism utilized by many tumor types to avoid T-cell response.^1–3^ The disruption of this interaction was targeted by many drug companies. Several immunotherapy antibodies (such as Opdivo, Keytruda, Tecentric, and Imfinzi) disrupting this interaction were FDA approved in recent years for treatment of Hodgkin’s lymphoma, melanoma, lung cancer, kidney cancer, bladder and other cancers.^1–3^ Previously we identified *miR-15/16*, key tumor suppressor microRNAs, as targets of 13q deletions in CLL.^4^ Loss of *miR-15/16* is the most common genetic lesion in CLL, promoting overexpression of BCL2 and causing CLL development. Since it was recently reported that *miR-16* target PD-L1 expression^5^ and regulate PD-1–PD-L1 interaction, we thought that *PDCD1* might be also be under *miR-15/16* control. Here we identified a 28 bp DNA fragment containing eight overlapping *miR-15/16* target sites in the 3’ UTR of *PDCD1*. Using luciferase assay and western blot analysis we demonstrated that *miR-15/16* target *PDCD1* expression. Since *miR-15/16* regulate PD-1 and PD-L1 expression, our results suggest that *miR-15/16* are critical in the regulation of PD-1–PD-L1 interaction, a critical mechanism utilized by malignant cells to avoid T-cell immunity (Fig. 1g). Restoration of *miR-15/16* activity in both, T-cells and tumor cells can be a promising opportunity in cancer therapy.

## Supplementary information

Supplementary data

## Data Availability

The datasets used and/or analyzed during this study are available from the corresponding author on reasonable request.
