# Supplementary material for: PDCD1 (PD-1) is a direct target of miR-15a-5p and miR-16-5p
Source: Signal Transduct Target Ther. 2022 Jan 19;7:12. doi: 10.1038/s41392-021-00832-9 (PMC8766454; doi:10.1038/s41392-021-00832-9)
Supplement: Supplementary file 1 — Supplementary data [file 41392_2021_832_MOESM1_ESM.docx]

Supplementary Materials for

*PDCD1* (PD-1) is a direct target of *miR-15a-5p* and *miR-16-5p*

Alexey Palamarchuk^1^, Liudmyla Tsyba^1^, Luisa Tomasello^1^, Yuri Pekarsky^1^^ and Carlo M. Croce^1^^

^1^Department of Cancer Biology and Genetics, Comprehensive Cancer Center, The Ohio State University, Columbus, OH 43210, USA

**^Corresponding author**s:

Carlo M. Croce, MD

Yuri Pekarsky, PhD

The Ohio State University, Comprehensive Cancer Center

Biomedical Research Tower, Room 1082

460 West 12th Avenue, Columbus, OH 43210, USA

[carlo.croce@osumc.edu](mailto:carlo.croce@osumc.edu)

pekarsky.yuri@osumc.edu

**Materials and Methods.**

**DNA Constructs.**

An expression construct containing untagged human *PDCD1* ORF and it’s full-length 3’-UTR (pCMV-*PDCD1*) was purchased from OriGene. For psiCHECK2-*PDCD1*-WT, a 496 bp-long fragment of *PDCD1* 3′-UTR (corresponding to positions 974-1469 of NCBI Reference Sequence NM_005018.3) containing eight predicted overlapping target sites for *miR-15a-5p/miR-16-5p* was cloned downstream of the renilla luciferase gene in the psiCHECK2 vector (Promega). Mutant construct psiCHECK2-*PDCD1*-MUT with 28-nt long deletion (corresponding to positions 1228-1255 of NCBI Reference Sequence NM_005018.3) that removed all predicted overlapping target sites for *miR-15a-5p/miR-16-5p* was produced by using QuikChange II XL site-directed mutagenesis kit (Agilent) following manufacturer’s instructions.

**Cell Cultures, Western blot assay and Luciferase Assay.**

HEK293 cells were cultured in RPMI 1640 medium (Sigma-Aldrich) supplemented with 10% FBS. Transfections were carried out with Lipofectamine 2000 (ThermoFisher Scientific). For Western blot experiment, cells were transfected with 2 μg of pCMV-*PDCD1* and 200 pmol of *pre-miR‐15a-5p*, *pre-miR‐16-5p*, *pre-miR-148a-3p* mimics or scrambled negative control 1 (N.C.1) from ThermoFisher Scientific. To obtain whole cell extracts, HEK293 cells were lysed in RIPA buffer and cell lysates were clarified by centrifugation. Antibodies used for Western blots were: anti-PD-1 (cat. #TA806806, OriGene) and anti-GAPDH (cat. #5174, Cell Signaling Technology). Western blot images were captured by LI-COR Biosciences Odyssey Infrared Imaging System using IRDye antibodies (Two-color 181 multiplex detection). Protein bands were normalized using LI-COR Image Studio software (Version 5.2). For luciferase assay, HEK293 cells were co-transfected with 250 ng of various psiCHECK2 vector constructs and 100 pmol of pre-miR mimics. Twenty-four hours after transfection, firefly and renilla luciferase activities were measured using the Dual-Luciferase Report Assay (Promega).

**RNA and quantitative real-time PCR (qRT-PCR).**

Total RNA was isolated from human cell lines using TRIzol (Invitrogen) according to manufacturer’s protocol. For qRT-PCR, TaqMan mRNA assays from ThermoFisher (*PDCD1* # Hs01550088_m1 and *GAPDH* # Hs02758991_g1, used as normalizer) as well as TaqMan miRNA assays (*hsa-miR-15a-5p* # 000389*, hsa-miR-16-5p* # 000391) were used to detect mRNAs and mature miRNAs expression, correspondingly. *RNU44* (ThermoFisher TaqMan assay # 00194) was used as normalizer for miRNA assays. All qRT-PCRs were carried out in triplicate using QuantStudio 12K Flex System (ThermoFisher).
